# Supplementary material for: The genome sequence of wall speedwell, Veronica arvensis L. (Lamiales: Plantaginaceae)
Source: Wellcome Open Res. 2026 Sep 18;11:706. [Version 1] doi: 10.12688/wellcomeopenres.27854.1 (PMC13620361; doi:10.12688/wellcomeopenres.27854.1)
Supplement: Peer review history

# Reviewer report: Reviewer response for version 1

**DOI:** 10.21956/wellcomeopenres.30651.r170082

**Published:** 2026-10-12

**Authors:** Samuel J. Smit

**Affiliations:** 1 University of York, York, England, UK

The author reports the assembly of a chromosome-level genome for *Veronica arvensis*, a member of the Plantaginaceae. All metrics evaluated indicate that the primary assembly is of high quality and complete. The assembly is furthermore annotated to a high standard with methods clearly described and all data made publicly accessible.

Are the datasets clearly presented in a usable and accessible format, and the assembly and annotation available in an appropriate subject-specific repository?

Yes

Are sufficient details of the sequencing and extraction, software used, and materials provided to allow replication by others?

Yes

Are the rationale for sequencing the genome and the species significance clearly described?

Yes

Are the protocols appropriate and is the work technically sound?

Yes

Reviewer Expertise:

plant genome assembly and comparative genomics

I confirm that I have read this submission and believe that I have an appropriate level of expertise to confirm that it is of an acceptable scientific standard.

# Reviewer report: Reviewer response for version 1

**DOI:** 10.21956/wellcomeopenres.30651.r170079

**Published:** 2026-10-05

**Authors:** Nicholas P. Tippery

**Affiliations:** 1 University of Wisconsin-Whitewater, Whitewater, USA

This is a fairly straightforward type of data to report, and everything appears to have been done according to standard practices. Veronica arvensis is an important species to have sequenced in this way. It might have been nice to have more evaluation of the genomic similarity to related species or some phylogenetic analyses, but I imagine these kinds of analyses will happen over time.

Are the datasets clearly presented in a usable and accessible format, and the assembly and annotation available in an appropriate subject-specific repository?

Yes

Are sufficient details of the sequencing and extraction, software used, and materials provided to allow replication by others?

Yes

Are the rationale for sequencing the genome and the species significance clearly described?

Yes

Are the protocols appropriate and is the work technically sound?

Yes

Reviewer Expertise:

Molecular phylogenetics, botany

I confirm that I have read this submission and believe that I have an appropriate level of expertise to confirm that it is of an acceptable scientific standard.

# Reviewer report: Reviewer response for version 1

**DOI:** 10.21956/wellcomeopenres.30651.r170084

**Published:** 2026-09-29

**Authors:** Dirk C. Albach

**Affiliations:** 1 Carl von Ossietzky-University, Oldenburg, Germany

This is the report of the genome of Veronica arvensis. The author has ample experience in reporting genomes and the manuscript is written in parallel to other genomes reported. Therefore, there is a certain routine in these papers. I only noticed that the results of the flow cytometry and DNA barcoding are not reported. 

 The reference Harap (2025) is included twice in the references.

Are the datasets clearly presented in a usable and accessible format, and the assembly and annotation available in an appropriate subject-specific repository?

Yes

Are sufficient details of the sequencing and extraction, software used, and materials provided to allow replication by others?

Yes

Are the rationale for sequencing the genome and the species significance clearly described?

Yes

Are the protocols appropriate and is the work technically sound?

Yes

Reviewer Expertise:

plant systematics

I confirm that I have read this submission and believe that I have an appropriate level of expertise to confirm that it is of an acceptable scientific standard.

# Reviewer report: Reviewer response for version 1

**Published:** 2026-09-18

**Authors:** Automated Benchmarking

**Affiliations:** 1 Wellcome Sanger Institute, Hinxton, UK

As this article is a Genome Note, Automated Benchmarking has been used to determine whether the reported assembly passes four defined checks established by the Earth BioGenome Project: the raw and assembled data are publicly available, the Earth BioGenome Project three-part metric is met, the k-mer completeness exceeds 90%, and the BUSCO score passes the stated threshold. For Veronica arvensis (daVerArve1), all four checks passed. Data available: pass. ENA study PRJEB78314, raw-read accessions ERR13420764, ERR13420765, ERR13420766, ERR13420767, and ERR13389751, and assembly accessions GCA_964211895.1 and GCA_964211885.1 are public. Earth BioGenome Project three-part metric: pass. Haplotype 1 is reported as 6.C.Q67, meeting the 6.C.Q40 reference standard. k-mer completeness: pass. The combined haplotype assemblies have 99.09% k-mer completeness, exceeding the Earth BioGenome Project target of 90%. BUSCO: pass. Haplotype 1, which is proposed as the reference assembly for this species, has 95.5% complete BUSCOs (90.3% single-copy and 5.2% duplicated). BUSCO software version: 5.5.0; lineage dataset: eudicots_odb10. It meets the plant-specific target of greater than 90% complete BUSCOs. This decision uses values reported in the Genome Note and public accession records.

Reviewer Expertise:

NA

I confirm that I have read this submission and believe that I have an appropriate level of expertise to confirm that it is of an acceptable scientific standard.
